# The Stimulated Glycolytic Pathway Is Able to Maintain ATP Levels and Kinetic Patterns of Bovine Epididymal Sperm Subjected to Mitochondrial Uncoupling

**DOI:** 10.1155/2017/1682393

**Published:** 2017-05-09

**Authors:** João D. A. Losano, Juan Fernando Padín, Iago Méndez-López, Daniel S. R. Angrimani, Antonio G. García, Valquiria H. Barnabe, Marcilio Nichi

**Affiliations:** ^1^Department of Animal Reproduction, Faculty of Veterinary Medicine, University of São Paulo, São Paulo, SP, Brazil; ^2^Department of Pharmacology, Faculty of Medicine, Autonomous University of Madrid, Madrid, Spain; ^3^Department of Medical Science, Faculty of Medicine Ciudad Real, University of Castilla-La Mancha, Ciudad Real, Spain; ^4^School of Veterinary Medicine and Animal Science, Avenue Orlando Marques de Paiva, No. 87, 05508-270 São Paulo, SP, Brazil

## Abstract

Studies have reported the importance of mitochondria in sperm functionality. However, for some species, the glycolytic pathway appears to be as important as oxidative phosphorylation in ATP synthesis and sperm kinetics. These mechanisms have not been fully elucidated for bovine spermatozoa. Therefore, the aim of this study was to evaluate the role of mitochondria and the glycolytic pathway in ATP synthesis, sperm movement patterns, and oxidative homeostasis of epididymal spermatozoa in bovine specimens. We observed that mitochondrial uncoupling with protonophores significantly reduced ATP levels. However, these levels were reestablished after stimulation of the glycolytic pathway. We verified the same pattern of results for sperm kinetic variables and the production of reactive oxygen species (ROS). Thus, we suggest that, after its appropriate stimulation, the glycolytic pathway is capable of maintaining ATP levels, sperm kinetic patterns, and oxidative balance of bovine epididymal spermatozoa submitted to mitochondrial uncoupling.

## 1. Introduction

Studies have shown the importance of mitochondria in sperm functionality, as they are considered the main source of ATP for cellular homeostasis and motility [1, 2]. However, the role of mitochondria in sperm metabolism has been a matter of debate. Mukai and Okuno [3] verified that ATP levels and flagellar beating remained constant when the mitochondria of mouse sperm was uncoupled concurrently with glycolysis stimulation. However, by inhibiting glycolysis and stimulating oxidative phosphorylation, authors observed that flagellar beating and ATP levels were quickly reduced. These results indicate that glycolysis plays an important role in murine sperm energy production.

In a similar study, Nascimento et al. [4] performed inhibitory and stimulatory treatments for both oxidative phosphorylation and glycolysis in human sperm. Authors concluded that oxidative phosphorylation, despite contributing to ATP production, is not sufficient to sustain sperm motility, confirming that the glycolytic pathway is the primary energy source for human sperm. Additionally, ATP produced by oxidative phosphorylation in the sperm midpiece is not efficiently released into the distal portions of the tail, indicating that glycolysis plays a key role in the flagellar beat of such sperm regions [5–7].

Davila et al. [8] demonstrated that equine spermatozoa require oxidative phosphorylation as glycolytic pathway to maintain motility. Complementary in ram, Losano et al. [9] demonstrated that mitochondrial depolarization did not affect total motility; however, sperm kinetic patterns were altered. On the other hand, they found that glycolytic pathway inhibition impaired total motility and sperm movement patterns. For both species, glycolytic pathway seems to be as important as oxidative phosphorylation for sperm physiology. However, the role of these pathways on bovine sperm functionality has not been fully elucidated. This information is extremely important for the understanding of bull sperm physiology. In addition, studies evaluating the energy metabolism of bovine sperm may contribute to the understanding of possible causes for the reduction in sperm quality and fertilization failures related to these metabolic pathways and then improve existent biotechnology's, such as artificial insemination which can impact in higher fertility rates.

Sperm collected directly from the epididymis seem to be the ideal cellular model to study energy metabolism. This is due to the many glycolysis, citric acid cycle, and oxidative phosphorylation stimulants contained in the seminal plasma derived from the accessory glands [10–12]. The fact that epididymal spermatozoa have not been stimulated with these substances provides a better in vitro manipulation of these cells, allowing the stimulation and inhibition of these pathways to evaluate the role of each metabolic pathway on sperm functionality.

Therefore, the aim of this study was to evaluate the role of mitochondria and glycolysis in ATP production, generation of reactive oxygen species (ROS), and kinetic patterns of epididymal bovine sperm by means of mitochondrial uncoupling and glycolytic pathway stimulation.

## 2. Material and Methods

The present experiment was conducted according to ethical guidelines for animal experiments and approved by the Bioethics Committee of the School of Veterinary Medicine and Animal Science at the University of São Paulo (protocol number 7978040914).

In this study, we submitted bovine epididymal spermatozoa to treatment with the oxidative phosphorylation uncoupler carbonyl cyanide 4-(trifluoromethoxy)phenylhydrazone (FCCP) to significantly reduce mitochondrial ATP synthesis and stimulated the glycolytic pathway by glucose addition. However, in order to verify the optimal concentrations of the uncoupler, FCCP, we performed a dose-response curve in experiment 1. Thus, the selected concentrations were used in the subsequent experiments. The aim of these experiments was to evaluate the contribution of mitochondria to ATP synthesis (experiment 2), patterns of sperm kinetics (experiment 3), and oxidative homeostasis (experiment 4) of bovine epididymal sperm and verify if stimulation of the glycolytic pathway would be able to maintain these sperm parameters that are probably suppressed by mitochondrial uncoupling.

### 2.1. Sample Collection

Epididymal sperm samples were collected and then dissecting the epididymis cauda with a scalpel blade, according to previous protocol [13]. To limit blood contamination, dissection was performed carefully. The flowing epididymal fluid was collected with an automatic pipette. Then, samples were used in each respective experiment proposed.

### 2.2. Experiment 1—Concentration-Response Curve of Mitochondrial Uncoupler, FCCP

To evaluate the effect of mitochondrial uncoupling by FCCP, spermatozoa from 3 bovine epididymides (*n* = 3) were collected and diluted to a concentration of 100 million spermatozoa per mL in modified TALP. Despite a minimum number of epididymis used, we evaluated the mitochondria of each spermatozoon singly, as experimental unit. Thus, we used 15 to 26 cells per FCCP concentration. Thereafter, the spermatozoa were incubated in a perfusion chamber with mitochondrial fluorophore tetramethylrhodamine-ethyl-ester perchlorate at 500 nM (ThermoFisher® Scientific, 0.5 *μ*L of TMRE in 1 mL of medium) for 5 minutes at 37°C. For the spermatozoa to remain attached during perfusion with FCCP, coverslips of the perfusion chamber were treated with polylysine.

After incubation, the amount of TMRE fluorescence captured by each sperm mitochondrion was recorded by the software LAS AF Lite (Leica® Microsystems, Germany) at an emission of 500 nm and excitation of 600 nm by a fluorescence microscope (Leica Microsystems, Germany). Thirty seconds of mitochondrial basal fluorescence was recorded, and then perfusions were performed with increasing FCCP concentrations (Tocris Bioscience®, MN, USA; 0.3, 1, 3, 10, 30, 60, and 100 *μ*M) by means of an electrovalve controller. Stimulation performed with FCCP at 30 seconds was recorded, and the percentage of mitochondrial depolarization was calculated based on the difference between the basal fluorescence and the amount of fluorescence retained in the mitochondria of each spermatozoon after 30 seconds of FCCP stimulation.

The lower FCCP concentrations of the dose-response curve (0.3, 1, and 3 *μ*M) and the concentration insufficient for the promotion of mitochondrial depolarization (0.1 *μ*M, concentration under the curve) were selected to be used in the subsequent experiments. We selected these concentrations in order to significantly reduce the mitochondrial ATP synthesis without promoting disruption in this organelle.

### 2.3. Experiment 2—Effect of Mitochondrial Uncoupling and Glycolysis Stimulation on ATP Levels

In this experiment, spermatozoa from 6 bovine epididymides (*n* = 6) were collected and diluted to a concentration of 100 million spermatozoa per mL in modified TALP medium. Each sample was divided into ten aliquots, which were submitted to a 5 × 2 factorial design wherein one of the factors was the addition of glucose (5 mM) and the other factor was the treatment with increasing concentrations of FCCP (0.1, 0.3, 1, and 3 *μ*M). After a 15-minute incubation, the treatments were subjected to measurements of ATP levels by means of a luminescence technique. For this procedure, 50 *μ*L aliquots in duplicate from each treatment containing 100000 spermatozoa were added to 50 *μ*L of CellTiter-Glo® Luminescent Cell Viability Assay kit (Promega®, USA) and incubated for 30 minutes at 37°C according to the manufacturer's recommendations. Immediately after this procedure, ATP levels were measured in a luminescence apparatus (ThermoFisher Scientific, MA, USA) in duplicate. The results obtained, expressed in arbitrary light units (AUL), were interpolated on a standard curve containing different concentrations of ATP (10, 100, 1000, 5000, and 10000 nM) and were then expressed in nM ATP.

### 2.4. Experiment 3—Effect of Mitochondrial Uncoupling and Glycolysis Stimulation on Sperm Kinetic Patterns

To evaluate the effect of mitochondrial uncoupling and glycolysis stimulation on sperm kinetic patterns, spermatozoa from 7 bovine epididymides (*n* = 7) were collected and diluted to a concentration of 100 million spermatozoa per mL in modified TALP medium. Each sample was divided into ten aliquots, which were submitted to a 5 × 2 factorial design wherein one of the factors was the addition of glucose (5 mM) and the other was the treatment with increasing concentrations of FCCP (0.1, 0.3, 1, and 3 *μ*M). After 5 minutes of incubation, the sperm samples were subjected to computerized analysis of sperm kinetics (ISASPBOS, Proiser®, Valencia, Spain). The following variables were considered: motility (%), progressive motility (%), VAP (average path velocity, *μ*m/s), VSL (straight-line velocity, *μ*m/s), VCL (curvilinear velocity, *μ*m/s), ALH (amplitude of lateral head displacement, *μ*m), BCF (beat cross-frequency, Hz), STR (straightness, %), and LIN (linearity, %). In addition to these parameters, the sperm were also divided into four groups based on velocity: rapid (VAP > 50 *μ*m/s; %), medium (30 *μ*m/s < VAP < 50 *μ*m/s; %), slow (VAP < 30 *μ*m/s or VSL < 15 *μ*m/s; %), and static (%) [14].

### 2.5. Experiment 4—Effect of Mitochondrial Uncoupling and Glycolysis Stimulation on Reactive Oxygen Species Production

To evaluate the effect of mitochondrial uncoupling and glycolysis stimulation on reactive oxygen species production, spermatozoa from 6 bovine epididymides (*n* = 6) were collected and diluted to a concentration of 100 million spermatozoa per mL in modified TALP. Each sample was divided into ten aliquots, which were submitted to a 4 × 2 factorial design wherein one of the factors was the addition of glucose (5 mM) and the other was the treatment with increasing concentrations of FCCP (0.1, 0.3, 1, and 3 *μ*M). These treatments were incubated for 30 minutes at 37°C and subjected to the detection of reactive oxygen species. To perform this technique, 100000 sperm were incubated in modified TALP solution containing 10 *μ*M (final concentration) of the fluorescent probe CM-H2DCFDA for 30 minutes (triplicate samples). After incubation was performed, the ROS were detected using a fluorometer (Fluostar microplate reader Omega, Labtec-BMG, Germany) at excitation 492–495 nm and emission 517–527 nm. The fluorescence intensity results obtained were interpolated on a standard curve containing different concentrations of hydrogen peroxide (H_2_O_2_: 3, 10, 30, 60, 100, 200, and 300 *μ*M) and were then expressed in *μ*L of O_2_ generated. Data were normalized relative to the control group (untreated samples).

### 2.6. Statistical Analysis

The concentration-response curve for FCCP (experiment 1) was performed by nonlinear regression using the software GraphPad Prism 6. Data relating to the measurement of ATP levels and computerized analysis of sperm kinetics (experiments 2 and 3, resp.) were analyzed using the SAS System for Windows (SAS Institute Inc., Cary, NC, USA). Thus, the interaction between FCCP and glucose factors was determined by PROC GLM. Differences between treatments were assessed using parametric (Student's *t*-test for each factor separately or LSD test for the combination of factors) and nonparametric tests (Wilcoxon) in accordance with the normality of the residuals (Gaussian distribution) and homogeneity of the variances. To analyze the effect of FCCP in the presence or absence of glucose in the ROS production, data normalized to the control group were compared by ANOVA variance analysis (LSD test) using the SAS System for Windows program (SAS Institute Inc., Cary, NC, USA). The level of significance to reject the H0 (null hypothesis) was 5%; that is, the significance level was 0.05. Significant differences between classificatory variables (treatments) and a specific response variable were considered.

## 3. Results

### 3.1. Experiment 1—Concentration-Response Curve of Mitochondrial Uncoupler FCCP

**Figure 1:**
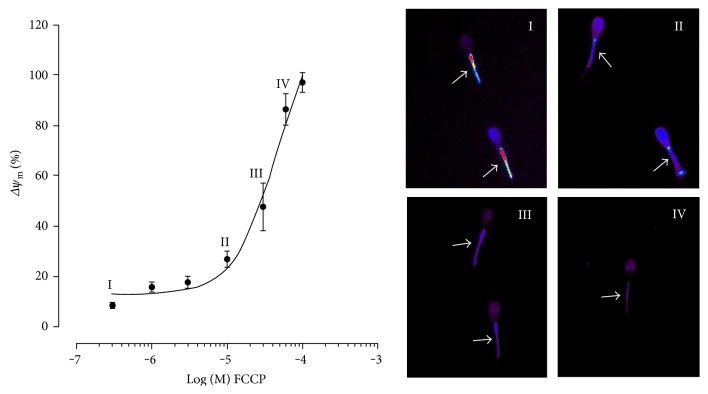
Dose-response curve of FCCP concentrations (0.3, 1, 3, 10, 30, 60, and 100 *μ*M) in sperm of bovine epididymal samples. Superscript numerals indicate the FCCP concentration used and their respective images. Mitochondrial depolarization of bovine spermatozoa at FCCP concentration of 0 *μ*M (I), 10 *μ*M (II), 30 *μ*M (III), and 60 *μ*M (IV). Arrows indicate mitochondria labeled with the TMRE fluorescent probe at different percentages of mitochondrial depolarization. 400x magnification.

By using a nonlinear regression, we found that the concentration-response curve is square root = 0.7 and EC50 = 4.67 × 10^−5^ *μ*M. We observed a high percentage of depolarization with FCCP concentrations of 30 *μ*M, 60 *μ*M, and 100 *μ*M (Figure 1). Thus, in order to select points where there is a reduction in ATP without promoting disruption in the organelle, we selected 3 *μ*M, 1 *μ*M, 0.3 *μ*M, and 0.1 *μ*M for the concentrations used in the subsequent experiments (concentration under the curve—Figure 1).

### 3.2. Experiment 2—Effect of Mitochondrial Uncoupling and Glycolysis Stimulation on ATP Levels

**Figure 2:**
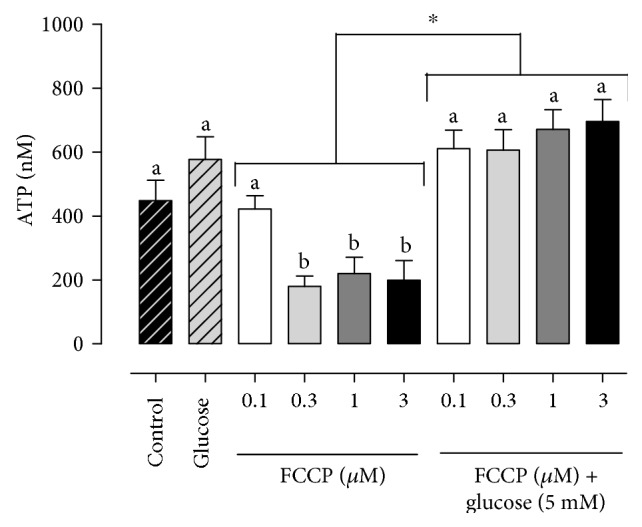
ATP production by bovine epididymal sperm treated with FCCP in different concentrations (0 *μ*M, 0.1 *μ*M, 0.3 *μ*M, 1 *μ*M, and 3 *μ*M) in absence or presence of glucose 5 mM. a-b superscripts indicate differences between concentrations (*P* < 0.05). ∗ indicates differences after the glucose supplementation (*P* < 0.05).

There were significant effects of FCCP, glucose, and FCCP-by-glucose interaction in the ATP (*P* < 0.0001; Table 1) analysis. Then, it was possible to compare the effects of the addition of glucose in the FCCP sample (Figure 2). We observed a lower ATP production in the FCCP group at concentrations of 0.3 *μ*M (180.3 ± 31.9 nM), 1 *μ*M (220.2 ± 40.4 nM), and 3 *μ*M (272.3 ± 70.4 nM) than at 0 *μ*M (control—448.6 ± 63.7 nM) and 0.1 *μ*M (422.4 ± 41.5 nM—Figure 2). However, in the group treated with FCCP supplemented with glucose, the concentrations were similar between the groups treated with 0.1 *μ*M (610.8 ± 57.8 nM), 0.3 *μ*M (606.2 ± 64.2 nM), 1 *μ*M (670.9 ± 61.9 nM), and 3 *μ*M (696.1 ± 68.5 nM) FCCP and the group treated with glucose without FCCP (577.2 ± 70.4 nM) (Figure 2).

### 3.3. Experiment 3—Effect of Mitochondrial Uncoupling and Glycolysis Stimulation on Sperm Kinetics Patterns

There were significant effects of FCCP, glucose, and FCCP-by-glucose interaction (*P* < 0.05) on all CASA parameters (Table 1).

**Figure 3:**
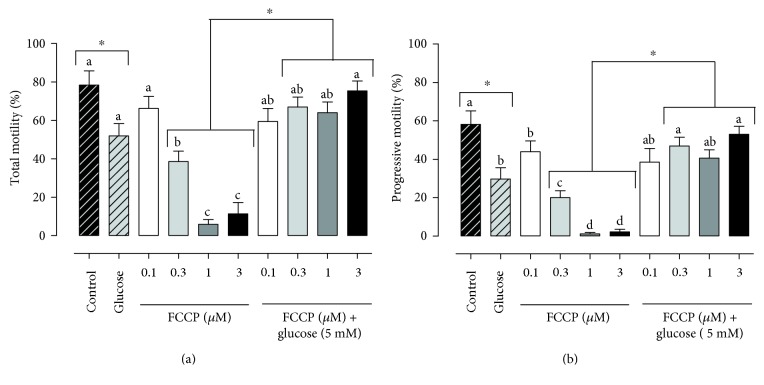
Total (a) and progressive (b) motility in bovine epididymal sperm treated with FCCP in different concentrations (0 *μ*M, 0.1 *μ*M, 0.3 *μ*M, 1 *μ*M, and 3 *μ*M) in absence or presence of glucose 5 mM. a–d superscripts indicate differences between concentrations (*P* < 0.05). ∗ indicates differences after the glucose supplementation (*P* < 0.05).

We observed a decrease in the total motility between samples without FCCP (control) and with glucose (Figure 3(a)); however, it was possible to note an increase in motility in the groups treated with 0.3 *μ*M, 0.1 *μ*M, 1 *μ*M, and 3 *μ*M FCCP supplemented with glucose (Figure 3(a)). This same effect was detected for progressive motility (Figure 3(b)), VAP, VSL, VCL, and rapid sperm velocity (see Supplementary Material available online at https://doi.org/10.1155/2017/1682393).

Next, we examined the effects of the addition of glucose in the FCCP samples (Figure 3 and Supplementary Material). In the BCF analysis, we observed an increase in the groups with 1 *μ*M and 3 *μ*M of FCCP supplemented with glucose but a decrease in the glucose group (Supplementary Material). Furthermore, we observed an increase in the slow sperm velocity in the samples supplemented with glucose in the groups treated with 1 *μ*M and 3 *μ*M of FCCP and glucose alone but a decrease in the group treated with 0.3 *μ*M FCCP (Supplementary Material).

With FCCP treatment, the control and 0.1 *μ*M groups had higher values of total sperm motility, VAP, and VSL than the 0.3 *μ*M group, which was superior to the 1 *μ*M and 3 *μ*M samples (Figure 3 and Supplementary Material). However, in the ALH, BCF, straightness, linearity, and wobble analyses, the control, 0.1 *μ*M, and 3 *μ*M groups had higher rates than the 1 *μ*M and 3 *μ*M groups (Supplementary Material). In the VCL and percentage of medium sperm velocity, we observed that the 3 *μ*M and 1 *μ*M groups had lower values than the 0.3 *μ*M group, which was similar to the 0.1 *μ*M group but lower than the control (Supplementary Material). In progressive motility (PM), the control group had the highest rates (Figure 3). However, we observed lower rates of PM in the 3 *μ*M and 1 *μ*M groups than in the 0.3 *μ*M group, which was inferior to the 0.1 *μ*M group (Figure 3). In the medium sperm velocity, the control group was superior to the 1 *μ*M and 3 *μ*M groups (Supplementary Material). On the other hand, in the slow sperm velocity, the control and 1 *μ*M groups had lower rates than the 0.1 *μ*M and 0.3 *μ*M groups (Supplementary Material).

When we compared the results between the concentrations of FCCP supplemented with glucose, we highlighted the higher values of progressive motility, straightness, and rapid sperm velocity in the groups treated with 3 *μ*M and 0.3 *μ*M of FCCP, which were superior to the glucose group (Figure 3 and Supplementary Material). In the total motility analysis, the 3 *μ*M group was superior to the glucose group (Figure 3). However, in the VCL, the 0.3 *μ*M group had higher values than the 1 *μ*M group (Supplementary Material). The glucose group was lower than the 0.3 *μ*M, 1 *μ*M and 3 *μ*M groups in the BCF parameter (Supplementary Material). However, in the slow sperm velocity, the 1 *μ*M group was higher than the 0.3 *μ*M group (Supplementary Material). The remaining CASA variables did not show any difference between the groups (Supplementary Material).

### 3.4. Experiment 4—Effect of Mitochondrial Uncoupling and Glycolysis Stimulation on Reactive Oxygen Species Production

**Figure 4:**
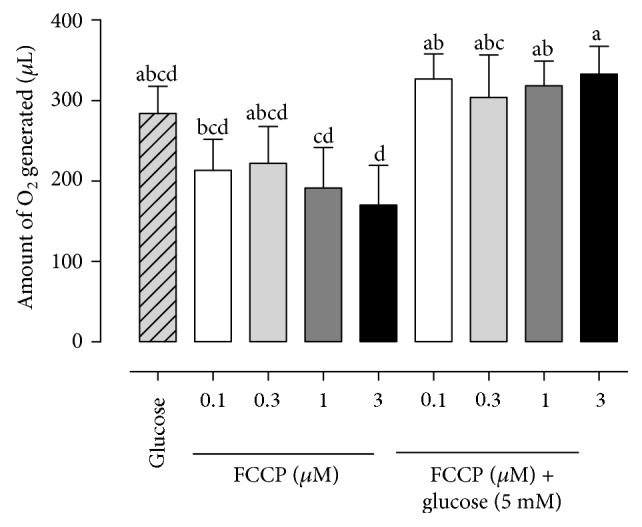
Amount of O_2_ generated by bovine epididymal sperm treated with FCCCP in different concentrations (0 *μ*M, 0.1 *μ*M, 0.3 *μ*M, 1 *μ*M, and 3 *μ*M) in absence or presence of glucose 5 mM. a–d superscripts indicate differences between concentrations (*P* < 0.05).

In the production of the reactive oxygen species, we highlight in Figure 4 the higher ROS generated by sperm treated with 3 *μ*M of FCCP supplemented with glucose (332.9 ± 34.58 *μ*L) than that with FCCP concentrations of 0.1 *μ*M (213.2 ± 38.77 *μ*L), 1 *μ*M (191.44 ± 50.39 *μ*L), and 3 *μ*M (170.06 ± 49.34 *μ*L).

## 4. Discussion

The aim of this study was to evaluate the role of mitochondria and the glycolytic pathway in the maintenance of ATP levels, the parameters of sperm movement, and the production of reactive oxygen species in epididymal bovine sperm. To perform this experiment, we submitted bovine sperm to mitochondrial uncoupling with FCCP to significantly reduce the synthesis of ATP by the mitochondria and evaluate the effect of this reduction in sperm functionality. Furthermore, we promoted stimulation of the glycolytic pathway by glucose addition concurrently with the mitochondrial uncoupling to assess whether glycolysis would be able to maintain the ATP levels, sperm kinetic patterns, and oxidative homeostasis possibly harmed by mitochondrial depolarization.

The mitochondrial uncoupler FCCP is a lipophilic molecule with protonophore properties; in other words, it is capable of interacting with the inner mitochondrial membrane to allow pumped protons to return to the mitochondrial matrix, dissipating the proton gradient and influencing the mitochondrial chemiosmosis [15, 16]. Indeed, in our experiment, we confirmed the depolarizing effect of the uncoupler FCCP. In experiment 2, we observed a significant reduction in ATP levels in the groups treated with 0.3, 1, and 3 *μ*M of FCCP compared to the control group. ATP production in the mitochondria occurs by means of the coupling of two reactions: the transport of electrons throughout the respiratory chain and the proton gradient. This latest gradient is capable of storing energy, called proton motive force, which drives the synthesis of ATP through ADP and inorganic phosphate [17]. FCCP has a protonophore effect that will dissipate the proton gradient, thereby reducing ATP synthesis, as noted in our results. On the other hand, the groups that were treated with these same FCCP concentrations but were supplemented with glucose had higher levels of ATP, similar to the control group. From these results, we can suggest that the glycolytic pathway, after being stimulated, is able to maintain ATP levels in bovine epididymal sperm. In fact, our results were consistent with a previous study in boars, which demonstrated that sperm mitochondria account for only 5% of energy production, while the glycolytic pathway contributes to 95% [18]. Additionally, species such as mice may use ATP from glycolysis and mitochondrial respiration depending on their biological conditions without changing sperm functionality or sperm ATP levels [19].

In experiment 3, we observed a very similar pattern of results as in experiment 2. The motility and spermatic movement patterns were affected by mitochondrial uncoupling. However, stimulation of the glycolytic pathway maintained sperm kinetic patterns, even with cells undergoing mitochondrial uncoupling. These results suggest that for bovine sperm, there is a close relationship between motility and ATP levels. However, this relationship is still a matter of controversy. In accordance with our study, Mukai and Okuno [3] verified that ATP levels and flagellar beating remained constant when mouse sperm mitochondria were uncoupled concurrently with the supplementation of substrates for glycolysis. Additionally, Krzyzosiak et al. [20] also observed that bovine sperm are capable of maintaining similar motility patterns in both aerobic and anaerobic conditions, assuming that glycolysis is capable of maintaining sperm motility. On the other hand, Ramió-Lluch et al. [21] demonstrated that the inhibition of ATP synthase impairs sperm motility, while intracellular ATP levels remain unchanged. Therefore, the author suggested an unknown essential mitochondrial mechanism responsible for motility maintenance that does not rely only on the maintenance of ATP levels. The variations in the results of the different experiments seem to be related to the species involved and the biological conditions to which such cells have been subjected [22, 23]. Therefore, there is a need for further studies to elucidate these mechanisms.

Regarding experiment 4, we observed that the groups treated with FCCP at 1 and 3 *μ*M in the absence of glucose had a lower production of reactive oxygen species (ROS). The reactive oxygen species produced by sperm play a key role in many physiological processes such as hyperactivation [24], capacitation [25], and the interaction between the sperm and oocyte [26]. The fact that the groups treated with FCCP and glucose did not differ from the control group suggests that glycolysis stimulation is able to maintain the physiological ROS production and, ultimately, oxidative balance. Moreover, the ability of FCCP in the absence of glucose to reduce ROS production reveals a possible therapeutic potential for preventing the release of excessive reactive oxygen species. This ability to prevent ROS production may be due to the increase of the electron transport rates accompanied by a reduction in mitochondrial intermediate states which is able to donate electrons to oxygen [27]. Furthermore, studies have demonstrated that the reduction in ATP synthesis by mitochondria is accompanied by a reduction in ROS production [28]. In fact, studies have shown this ability of mitochondrial uncouplers in somatic cells [29, 30].

Therefore, knowledge of the role of each metabolic pathway on sperm functionality may target therapies using substrates to stimulate these pathways in reproduction biotechnologies. Furthermore, the data of mitochondrial uncoupling FCCP reduces the reactive oxygen species production and suggests that this molecule can be used to prevent seminal oxidative stress during procedures that induce this stress, such as cryopreservation. Thus, such procedure can improve reproductive indexes by means of assisted reproduction techniques in cattle, especially intrauterine artificial insemination. Nevertheless, this therapeutic effect should be further studied in spermatozoa.

## 5. Conclusion

In conclusion, after its stimulation, the glycolytic pathway is capable of maintaining ATP levels, sperm kinetic patterns, and oxidative balance of bovine epididymal spermatozoa submitted to mitochondrial uncoupling.

## Supplementary Material

Supplementary material – Sperm kinetics patterns, ATP levels and ROS detection (amount of O2 generated) of bovine epididymal sperm treated with FCCCP in different concentrations (0µM, 0.1µM, 0.3µM, 1µM and 3µM) in absence or presence of glucose 5mM.

## Figures and Tables

**Table 1:** Probability values for the FCCP (0, 0.1, 0.3, 1, and 3 *μ*M), glucose, and their interaction on computer-assisted sperm analysis (CASA).

	FCCP	Glucose	FCCP × glucose
Total sperm motility (%)	<0.0001	0.0003	<0.0001
Sperm progressive motility (%)	<0.0001	0.0005	<0.0001
Percentage of rapid sperm (%)	0.0006	0.0077	<0.0001
Percentage of medium sperm (%)	0.0087	0.0033	<0.0001
Percentage of slow sperm (%)	0.3993	0.0361	0.0045
Amplitude of lateral head displacement (ALH—*μ*m)	0.0009	0.0119	0.0095
Average path velocity (VAP—*μ*m/s)	<0.0001	0.0002	<0.0001
Straight line velocity (VSL—*μ*m/s)	<0.0001	0.0002	<0.0001
Curvilinear velocity (VCL—*μ*m/s)	0.0002	0.0038	0.0002
Beat cross-frequency (BCF—Hz)	<0.0001	0.0020	<0.0001
Sperm straightness (STR—%)	0.0002	0.0020	<0.0001
Sperm linearity (LIN—%)	<0.0001	0.0003	<0.0001
Wobble (WOB—%)	<0.0001	0.0003	<0.001

## Abbreviations

ALH: Amplitude of lateral head displacement
ATP: Adenosine triphosphate
BCF: Beat cross-frequency
CASA: Computer-assisted sperm analysis
FCCP: Carbonyl cyanide 4-(trifluoromethoxy) phenylhydrazone
LIN: Sperm linearity
LSD: Least significant difference
ROS: Reactive oxygen species
STR: Sperm straightness
TALP: Tyrode albumin lactate pyruvate
VAP: Average path velocity
VCL: Curvilinear velocity
VSL: Straight line velocity
WOB: Wobble.
