# Supplementary material for: The Stimulated Glycolytic Pathway Is Able to Maintain ATP Levels and Kinetic Patterns of Bovine Epididymal Sperm Subjected to Mitochondrial Uncoupling
Source: Oxid Med Cell Longev. 2017 May 9;2017:1682393. doi: 10.1155/2017/1682393 (PMC5446860; doi:10.1155/2017/1682393)
Supplement: Supplementary file 1 — Supplementary material – Sperm kinetics patterns, ATP levels and ROS detection (amount of O2 generated) of bovine epididymal sperm treated with FCCCP in different concentrations (0µM, 0.1µM, 0.3µM, 1µM and 3µM) in absence or presence of glucose 5mM. [file 1682393.f1.docx]

**Supplementary material** – Sperm kinetics patterns, ATP levels and ROS detection (amount of O_2_ generated) of bovine epididymal sperm treated with FCCCP in different concentrations (0µM, 0.1µM, 0.3µM, 1µM and 3µM) in absence or presence of glucose 5mM.

|  |  | | | **SPERM KINETICS PATTERS** | | | | | | | | | |  | |  | |
| --- | --- | --- | --- | --- | --- | --- | --- | --- | --- | --- | --- | --- | --- | --- | --- | --- | --- |
|  |  | | **FCCP (µM)** | | | | |  | **FCCP (µM) + glucose (5mM)** | | | | | | | | |
|  | Control | 0.1µM | | | 0.3µM | 1µM | 3µM | | |  | Glucose | 0.1µM | 0.3µM | | 1µM | | 3µM |
| **Motility (%)** | 78.4±7.4^a^ | 66.3±6.2^a^ | | | 38.6±5.4^b^ | 5.9±2.5^c^ | 11.4±5.8^c^ | | |  | 51.9±6.4^b*^ | 59.4±6.8^ab^ | 67±5.1^ab*^ | | 64±5.6^ab*^ | | 75.4±5.1^a*^ |
| **Progressive (%)** | 58.23±7^a^ | 43.9±5.6^b^ | | | 20±3.6^c^ | 1.2±0.7^d^ | 2.2±1.4^d^ | | |  | 29.7±5.9^b*^ | 38.5±7.1^ab^ | 46.9±4.6^a*^ | | 40.6±4.3^ab*^ | | 53±4.2^a*^ |
| **VAP (µm/s)** | 50.6±3.3^a^ | 41.6±2.8^a^ | | | 30.5±3.8^b^ | 7.4±2.6^c^ | 8.4±3^c^ | | |  | 40.9±2.5^*^ | 40±4.6 | 46.7±2.7^*^ | | 41.6±2.8^*^ | | 45.9±1.2^*^ |
| **VSL (µm/s)** | 43.4±3.3^a^ | 35.6±3.6^a^ | | | 25.4±3.5^b^ | 3.6±1.6^c^ | 4.9±2^c^ | | |  | 32.1±2.9^*^ | 33.9±4.8 | 40.6±2.9^*^ | | 35.7±1.9^*^ | | 39.8±0.8^*^ |
| **VCL (µm/s)** | 70.6±1.6^a^ | 56.7±3.1^ab^ | | | 46.8±1.2^b^ | 23.2±8.3^c^ | 24.8±9.9^c^ | | |  | 56.5±2^ab*^ | 57.3±4.1^ab^ | 63.7±2.6^a*^ | | 54.5±2.3^b*^ | | 62.1±2.8^ab*^ |
| **ALH (µm)** | 3.0±6.1^a^ | 2.7±0.1^a^ | | | 2.7±0.2^a^ | 1.1±0.5^b^ | 1.1±0.5^b^ | | |  | 2.7±0.1 | 2.9±0.1 | 2.9±0.0 | | 2.5±0.1^*^ | | 2.8±0.2^*^ |
| **BCF (Hz)** | 2.8±0.2^a^ | 3±0.1^a^ | | | 2.4±0.1^a^ | 0.6±0.3^b^ | 0.8±0.5^b^ | | |  | 2.2±0.1^b*^ | 2.6±0.1^ab^ | 2.8±0.2^a^ | | 2.9±0.1^a*^ | | 2.8±0.1^a*^ |
| **STR (%)** | 85.4±1.7^a^ | 84.6±3.3^a^ | | | 82.4±1.6^a^ | 27.3±11.4^b^ | 33.5±14^b^ | | |  | 78.5±4.5^b^ | 83±2.7^ab^ | 86.8±1.6^a^ | | 86.2±1.8^ab*^ | | 86.9±2.4^a*^ |
| **LIN (%)** | 61.2±3.9^a^ | 62.9±5.4^a^ | | | 53.7±6.6^a^ | 9.1±4^b^ | 13.9±7^b^ | | |  | 55.6±4 | 57.3±4.5 | 63.4±2.3 | | 65.4±2.4^*^ | | 64.8±3.2^*^ |
| **RAP (%)** | 35±4.8^a^ | 24.2±4.7^b^ | | | 9.1±1.6^c^ | 0.8±0.8^c^ | 1.2±1.1^c^ | | |  | 13.4±1.6^b*^ | 20.8±4.8^ab^ | 26.5±3.3^a*^ | | 19±4.7^ab*^ | | 28.5±3.4^a*^ |
| **MED (%)** | 38.3±6.6^a^ | 30.6±6.4^ab^ | | | 18.5±4.1^b^ | 2.0±1^c^ | 2.9±1.5^c^ | | |  | 27.4±6 | 29.1±5.1 | 27.4±5.8 | | 32.5±7.2^*^ | | 38.8±4.9^*^ |
| **SLOW (%)** | 5.2±0.4^b^ | 11.3±1.3^a^ | | | 11.4±0.9^a^ | 3±1.2^b^ | 7±3.8^ab^ | | |  | 11.1±1.6^ab*^ | 9.9±0.9^ab^ | 8±0.9^b*^ | | 12.5±1.2^a*^ | | 10.2±1^ab^ |
| **WOB (%)** | 71.4±3.7^a^ | 75.2±3.6^a^ | | | 64.6±7.2^a^ | 18.4±6.5^b^ | 21.8±8.7^b^ | | |  | 72.4±3.6 | 68.5±3.7 | 72.9±1.5 | | 76±2.5^*^ | | 74.3±1.7^*^ |
| **ATP (nM)** | 448.6±63.7^a^ | 422.4±41.5^a^ | | | 180.3±31.9^b^ | 220.2±50.4^b^ | 272.3±70.4^b^ | | |  | 577.2±70.4^a^ | 610.8±57.8^a*^ | 606.2±64.2^a*^ | | 670.9±61.9^a*^ | | 696.1±68.5^a*^ |
| **ROS (µl O_2)_** | ---------- | 213.2±38.7^ab^ | | | 221.7±46.2^abc^ | 191.4±50.3^ab^ | 170±49.4^a^ | | |  | 283.9±33.8^abcd^ | 326.6±31.3^bcd^ | 303.8±52.7^abcd^ | | 318.3±30.6^cd^ | | 332.9±34.5^d^ |

^a-d Superscripts indicates differences between concentrations (P < 0.05). * Indicates differences after the glucose supplementation (P<0.05).. Motility – Total Sperm Motility; Progressive – Progressive Motility; VAP – Average path velocity; VSL – Straight line velocity; VCL – Curvilinear velocity; ALH – Amplitude of lateral head displacement; BCF – Beat cross-frequency; STR – Straightness; LIN – Linearity; RAP - Percentage of rapid sperm, MED - Percentage of medium sperm; SLOW - Percentage of slow sperm; WOB - Wobble. ATP –ATP levels (nM), ROS – ROS detection (µL)^
